# Magnesium transporter protein solute carrier family 41 member 1 suppresses human pancreatic ductal adenocarcinoma through magnesium-dependent Akt/mTOR inhibition and bax-associated mitochondrial apoptosis

**DOI:** 10.18632/aging.101940

**Published:** 2019-05-08

**Authors:** Jing Xie, Chien-shan Cheng, Xiao Yan Zhu, Ye Hua Shen, Li Bin Song, Hao Chen, Zhen Chen, Lu Ming Liu, Zhi Qiang Meng

**Affiliations:** 1 Department of Integrative Oncology, Fudan University Shanghai Cancer Center, Shanghai 200032, P. R. China; 2 Department of Oncology, Shanghai Medical College, Fudan University, Shanghai 200032, P. R. China

**Keywords:** SLC41A1, pancreatic ductal adenocarcinoma, apoptosis, Akt/mTOR, mitochondira membrane potential, ROS, aging, age-related disease

## Abstract

The aim of this study was to identify the function of the Mg^2+^ transporter protein solute carrier family 41 member 1 SLC41A1 in pancreatic ductal adenocarcinoma and the underlying mechanisms. A total of 27 solute carrier proteins were differentially expressed in pancreatic ductal adenocarcinoma. Three of these proteins were correlated with clinical outcomes in patients, among which SLC41A1 was downregulated in tumour. Overexpression of SLC41A1 suppressed orthotopic tumour growth in a mouse model and reduced the cell proliferation, colony formation, and invasiveness of KP3 and Panc-1 cells, which may have been associated with the increased population of apoptotic-prone cells. Overexpression of SLC41A1 reduced the mitochondrial membrane potential, induced Bax while suppressed Bcl-2 expression. Suppression of Bax abrogated the tumour-suppressive effects of SLC41A1. Furthermore, overexpression of SLC41A1 promoted Mg^2+^ efflux and suppressed Akt/mTOR activity, which is the upstream regulator of Bax and Bcl-2. An increase in Akt activity and supplementation with Mg^2+^ abolished SLC41A1-induced tumour suppression. The results of this study suggest that SLC41A1 may be a potential target for the treatment of pancreatic ductal adenocarcinoma.

## INTRODUCTION

Pancreatic cancer is one of the most aggressive human cancers of the gastrointestinal system [1]. The most common type of pancreatic cancer is pancreatic ductal adenocarcinoma (PDAC), which accounts for about 85% of all malignant pancreatic tumours [2]. There has been a rise in the number of deaths caused by PDAC, especially in developed countries. In 2015, it was ranked as the fourth most common cancer in the United States and was projected to be the second most common cancer by 2030 [3]. Early diagnosis of this disease remains very difficult, as symptoms do not usually appear in the early stages [4], resulting in the poor prognosis of PDAC. The 5-year survival rate of PDAC was only about 2% with a median survival of about four months [5]. Treatment of PDAC is limited; the best option is surgery, which improves 5-year survival to 20% but is still far from satisfactory [6]. Thus, it is important to identify novel diagnostic and therapeutic targets for PDAC.

The solute carrier (SLC) protein superfamily is a series of carrier proteins that mediate the transport of organic and inorganic substrates across the cell membrane. Members of the SLC family exhibit differences in substrate specificity, and predominantly facilitate the transport of particular substrates [7]. SLC proteins are ubiquitously expressed in the tissues and organs of the gastrointestinal tract, where they maintain physiological functions such as nutrient uptake, ion transport, as well as waste and toxin removal [8]. In recent years, there has been increasing interest in the diagnostic and therapeutic potential of SLC proteins, as research has shown that their abnormal expression was associated with the incidence and progression of both rare and common diseases [9]. In particular, the role of SLC proteins as diagnostic and therapeutic targets of human cancers was recently highlighted, with the belief that their aberrant expression may be responsible for nutrient and ion transport to meet the needs of proliferating tumour cells, as well as for drug elimination and chemoresistance [10, 11]. In PDAC, SLC proteins were thought to be potential predictive biomarkers that play a role in the mechanism of acquired drug resistance to chemotherapy [12]. However, the role of the magnesium (Mg^2+^) transporter SLC family 41 member 1 (SLC41A1) protein in the progression of PDAC has rarely been studied.

In this study, we identified the vital role of SLC proteins in PDAC progression and investigated the possible mechanisms involved. We retrieved human PDAC data from the Gene Expression Omnibus (GEO) database and screened differentially expressed SLC proteins in PDAC, with significant emphasis on patient survival. We found that SLC41A1 was downregulated in PDAC and had tumour-suppressive effects. *In vitro* and *in vivo* studies verified the function of SLC41A1 as a tumour suppressor. Cellular mechanisms associated with different signalling pathways were also investigated.

## RESULTS

### SLC proteins exhibit different expression patterns in PDAC

**Figure 1:**
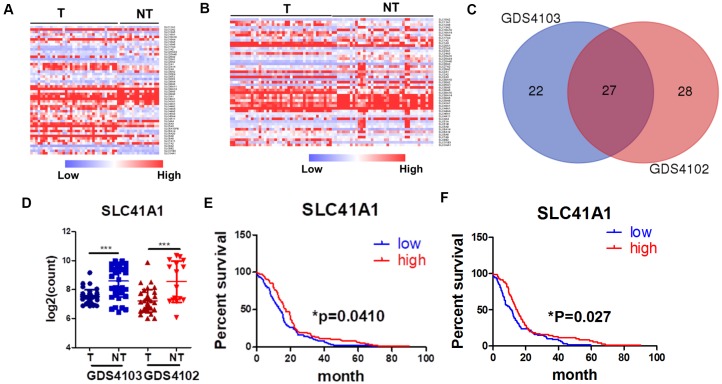
**SLC proteins are differentially expressed in PDAC.** (**A**) Expression profile of SLC proteins in dataset GDS1403. (**B**) Expression profile of SLC proteins in dataset GDS1402. (**C**) Venn diagram of overlapping genes between two datasets. (**D**) SLC41A expression was down-regulated in PDAC patients; (**E**) SLC41A expression was inversely correlated to the overall survival of PDAC patients; (**F**) SLC41A expression was inversely correlated to the progression-free survival of PDAC patients; *p < 0.05; ***p < 0.0o1.

The role of SLC proteins in pancreatic cancers has recently garnered increasing attention. In their review, Lemstrová and colleagues [13] postulated that SLC proteins might have prognostic and therapeutic potential in PDAC [12]. To determine the clinically relevant role of SLC proteins in PDAC, we retrieved transcriptomic expression profiles of SLC proteins from the GEO database. Heatmaps were generated by comparing SLC protein expression in PDAC tissues with that in non-tumour pancreatic tissues using two datasets, namely, GDS4103 [14] (Figure 1A) and GDS4102 [15] (Figure 1B). A total of 49 and 55 SLC proteins were differentially expressed in PDAC tissues from the GDS4103 and GDS4102 datasets, respectively, with statistical significance. The two datasets shared 27 SLC proteins, with expression patterns that were consistent across the different datasets (Figure 1C). Among these 27 proteins, 13 were overexpressed in PDAC compared with non-tumour tissues (Supplementary Figure 1), while 14 were downregulated (Supplementary Figure 2, Figure 1D).

### SLC41A1 expression is associated with the prognosis and staging of PDAC

To further understand the clinical role of differentially expressed SLC proteins in PDAC, we retrieved data from The Cancer Genome Atlas database to determine whether there was a correlation between SLC protein expression and patient survival. The preliminary screen used the median expression of the particular SLC proteins in a group of patients with PDAC and plotted the months of overall survival (OS) in a Kaplan–Meier plot. Of the 13 SLC proteins that were upregulated in PDAC, SLC6A14, SLC16A4, and SLC4A11 were statistically correlated with the OS of PDAC patients, according to the Kaplan–Meier plot (Supplementary Figure 3). While the high expression of SLC6A14 and SLC16A4 predicted a poor prognosis, it was contradictory to observe that the high expression of SLC4A11 predicted better survival. Of the 14 SLC proteins that were downregulated in PDAC, SLC16A10, SLC39A8, and SLC41A1 were statistically correlated with the OS of PDAC patients, according to the Kaplan–Meier plot (Supplementary Figure 4, Figure 1E); however, only SLC41A1 was consistently predicted that its high expression correlated with a better prognosis. To further identify critical factors of SLC proteins in PDAC, we plotted the disease-free survival data of SLC6A14, SLC16A4, and SLC41A1, as their expression patterns consistently correlated with the OS patterns. Both SLC6A14 and SLC41A1 predicted disease-free survival with statistical significance (Figure 1F, Supplementary Figure 5A). SLC6A14 was recently identified as a novel druggable target in PDAC [16, 17], suggesting the validity of our screening results, whereas the role of SLC41A1 has rarely been studied in cancer. SLC41A1 expression gradually decreased with the developing stages of PDAC (Supplementary Figure 5B) and was inversely associated with the presence of diabetes mellitus, one of the significant risk factors for PDAC (Supplementary Figure 5C). These results suggest that the clinical relevance of SLC41A1 in PDAC; thus, further biomedical investigations into this protein are warranted.

### Overexpression of SLC41A1 suppresses tumour growth in a mouse model of orthotopic PDAC

**Figure 2:**
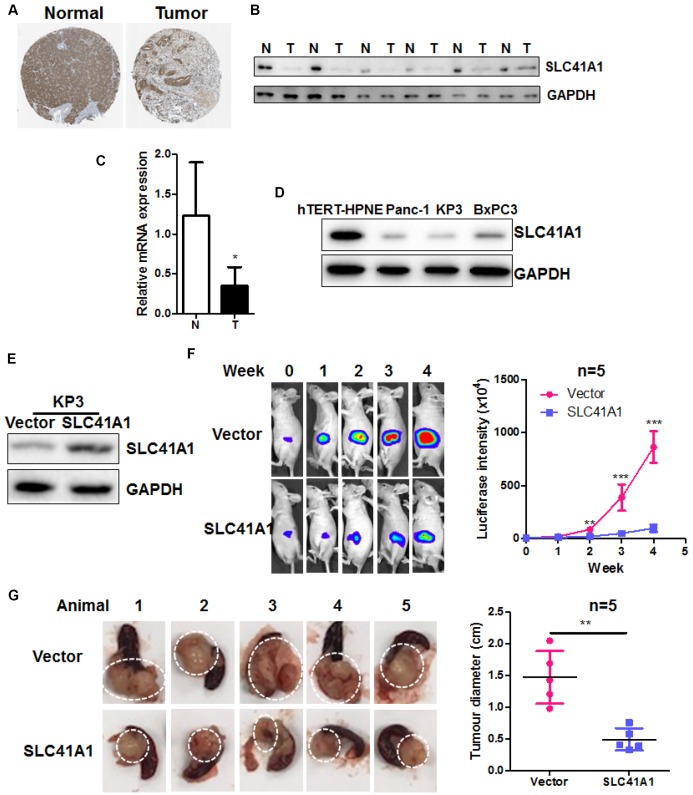
**Overexpression of SLC41A1 suppresses tumour growth in an orthotopic mouse model of PDAC**. (**A**) SLC41A1 protein was downregulated in human PDAC tissue. (**B**) SLC41A1 protein expression was suppressed in six PDAC tissue samples compared with paired non-adjacent pancreatic tissue. (**C**) SLC41A1 mRNA expression was suppressed in six PDAC tissues compared with paired non-adjacent pancreatic tissue. (**D**) Expression of SLC41A1 was downregulated in the human PDAC cell lines KP3, Panc-1, and BxPC3 compared with the normal pancreatic ductal epithelial cell line hTERT-HPNE. (**E**) Successful overexpression of SLC41A1 in KP3 cells that were used to create the orthotopic model (n = 5). (**F**) Overexpression of SLC41A1 suppressed the growth rate of orthotopic tumours in mice. (**G**) Tumour size was reduced by SLC41A1 overexpression. **p < 0.01; ***p < 0.001.

The function of SLC41A1 was recently studied in neurodegeneration such as Parkinson’s diseases [18–23]; however, its role in cancer, especially PDAC, was yet to be identified. We found that in human tissues of PDAC, the protein expression of SLC41A1 was significantly suppressed (Figure 2A, data retrieved from the Human Protein Atlas Project). Suppressed protein and mRNA expression of SLC41A1 was validated in six pairs of human PDACs and adjacent non-tumour tissues (Figure 2B, 2C). SLC41A1 expression was also downregulated in the human PDAC cell lines KP3, Panc-1, and BxPC3 compared with the normal pancreatic ductal epithelial cell line hTERT-HPNE (Figure 2D). To identify the role of SLC41A1 in PDAC, we overexpressed the protein in KP3 cells (Figure 2E). This cell line was tagged with luciferase to facilitate the non-invasive *in vivo* monitoring of tumour growth. Wild-type KP3 cells and the SLC41A1-overexpressing line KP3/SLC41A1 were then injected into the pancreas of athymic nude mice to create the orthotopic PDAC model. The growth of pancreatic tumours was imaged weekly. The overexpression of SLC41A1 in KP3 cells reduced the growth rate of pancreatic tumours, as evidenced by the smaller increase in luciferase signal (Figure 2F). At the end of the experiment, we dissected the pancreatic tumours from the mice and found that the pancreatic tumour size was significantly reduced in those injected with KP3/SLC41A1 cells (Figure 2G). These observations suggest that SLC41A1 has a tumour-suppressive role in PDAC.

### SLC41A1 is inversely correlated with the in vitro proliferation and invasion of human PDAC cells

**Figure 3:**
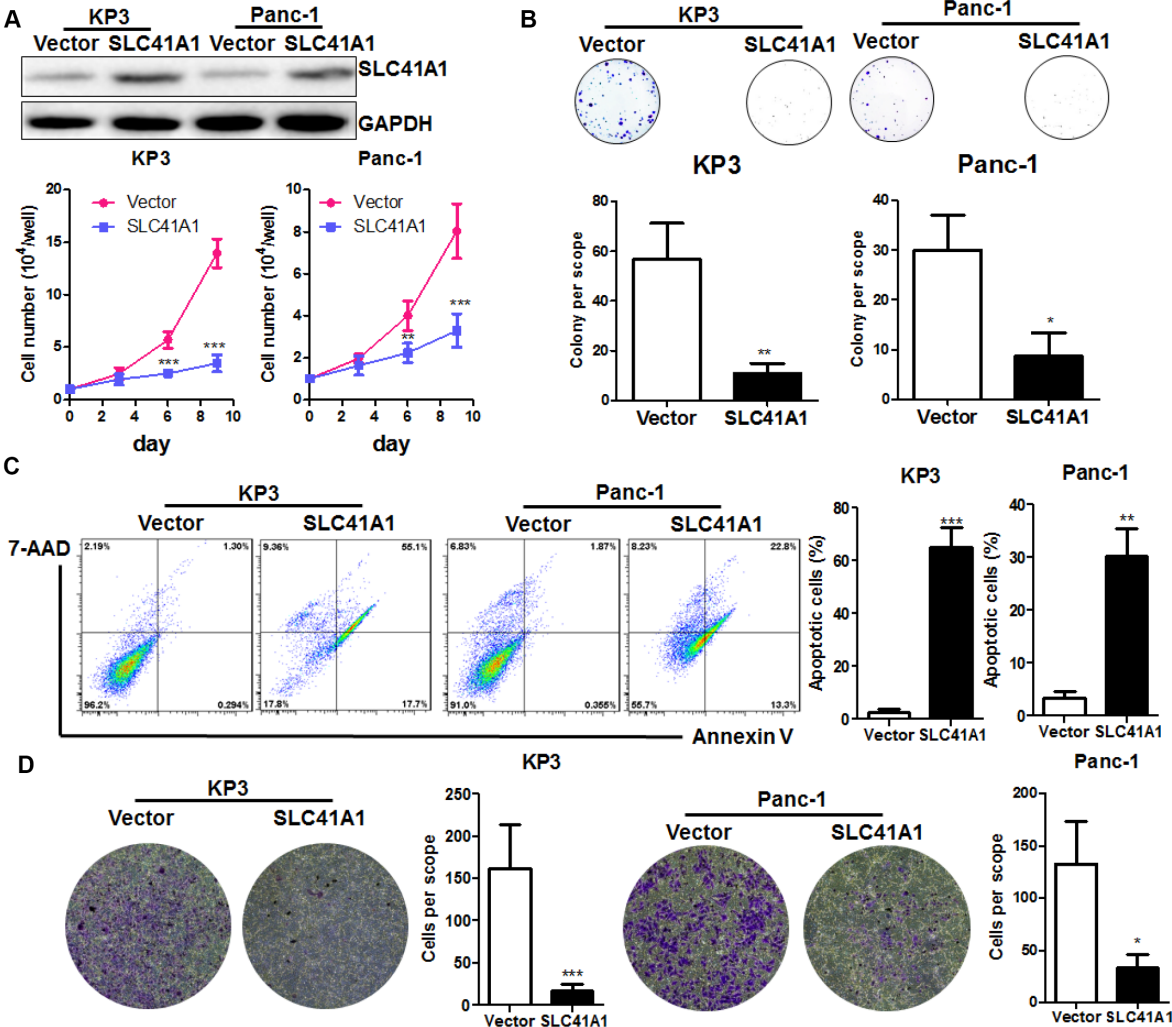
**Overexpression of SLC41A1 suppresses *in vitro* PDAC cell proliferation and induces apoptosis**. (**A**) Overexpression of SLC41A1 reduced the growth rate of PDAC cells KP3 and Panc-1. (**B**) Overexpression of SLC41A1 suppressed colony formation. (**C**) SLC41A1 induced the formation of apoptotic-like PDAC cells. (**D**) SLC41A1 suppressed the invasion of PDAC cells through the extracellular matrix. *p < 0.05; **p < 0.01; ***p < 0.001.

To further understand the role of SLC41A1 as a tumour suppressor in PDAC, we used a non-viral plasmid encoding the ORF of SLC41A1 to overexpress the SLC41A1 protein in parallel with the Panc-1 pancreatic tumour cell line. Overexpression of SLC41A1 significantly reduced the proliferation of both KP3 and Panc-1 cells (Figure 3A) and had reduced colony formation capacity (Figure 3B). To determine whether SLC41A1 initiated the apoptosis of PDAC cells, we performed Annexin-V/7-ADD staining. Flow cytometry analysis revealed that the proportion of apoptotic cells was markedly increased in PDAC cells with overexpression of SLC41A1, indicating that cells with high SLC41A1 expression were prone to apoptotic cell death (Figure 3C). Furthermore, overexpression of SLC41A1 remarkably reduced the invasion of PDAC cells through the extracellular matrix (Figure 3D). These observations, together with the results from our *in vivo* study, suggest that SLC41A1 may suppress PDAC cell proliferation, growth, and invasion by potentiating the cells towards the apoptotic phenotype.

### Bax protein-associated MMP loss contributes to SLC41A1-induced apoptosis in PDAC cells

**Figure 4:**
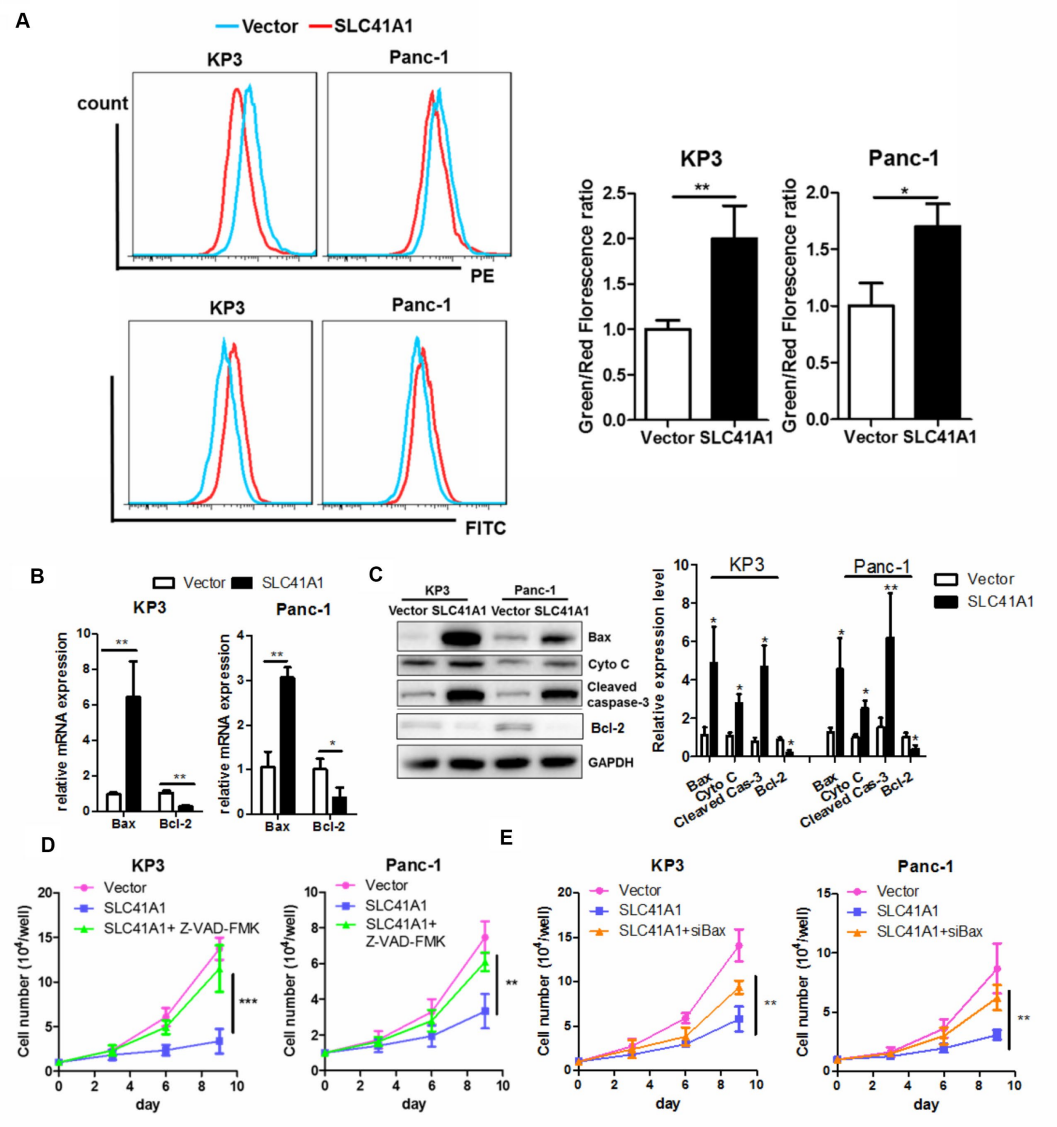
**Bax-associated mitochondrial apoptosis is involved in the tumour-suppressive effects of SLC41A1.** (**A**) Overexpression of SLC41A1 reduced the MMP, as indicated by JC-1 staining. (**B**) SLC41A1 activated Bax transcription and suppressed Bcl-2 transcription. (**C**) SLC41A1 increased Bax protein expression, cytochrome c release and cleavage of caspase-3, and reduced Bcl-2 protein level in PDAC cells. (**D**) Treatment of cells with a caspase inhibitor abrogated the tumour-suppressive effects of SLC41A1. (**E**) RNA interference of Bax abolished the tumour-suppressive effects of SLC41A1. *p < 0.05; **p < 0.01; ***p < 0.001.

While cell apoptosis mainly relies on activation of the extrinsic pathway upon binding of death receptors with their ligands such as Fas, there is an intrinsic apoptotic pathway that involves loss of MMP [24]. Because SLC41A1 overexpression shifted PDAC cells towards the pro-apoptotic phenotype in the presence of ectopic death ligands, we examined whether MMP loss was induced upon SLC41A1 expression. JC-1 staining was used to measure the MMP; strong red fluorescence suggested intact mitochondrial membrane integrity while an increased green signal indicated MMP loss [25]. Both KP3 and Panc-1 exhibited increased green/red fluorescence signal upon overexpression of SLC41A1, revealing mitochondrial membrane integrity (Figure 4A). The Bax protein, whose expression is induced during initiation and activation of the intrinsic apoptotic pathway, induces permeability of the outer mitochondrial membrane and causes loss of the MMP [26]. Increased Bax mRNA expression was observed in PDAC cells with SLC41A1 overexpression (Figure 4B). This was in accordance with our observation that Bax protein expression, as well as the release of Cytochrome C from mitochondria, was initiated upon SLC41A1 overexpression (Figure 4C). Reversed expression pattern of the anti-apoptotic counteract of Bax, Bcl-2, was also observed (Figure 4B, 4C). The release of Cytochrome C into the cytoplasm caused cleavage and activation of caspase-3, the final executor of apoptosis (Figure 4C). Addition of the caspase inhibitor Z-VAD-FMK (50 μM) significantly attenuated the proliferation inhibition induced by SLC41A1 overexpression in PDAC cells (Figure 4D), indicating that SLC41A1-associated switch towards the apoptotic phenotype was important in mediating the tumour-suppressive functions of the protein. Furthermore, silencing of Bax protein by RNA interference reduced the tumour-suppressive effects of SLC41A1, suggesting that Bax activation of the intrinsic apoptotic pathway mediates the tumour-suppressive effects of SLC41A1 (Figure 4E).

### SLC41A1-mediated efflux of cellular Mg^2+^ suppresses activation of Akt/mTOR signalling

**Figure 5:**
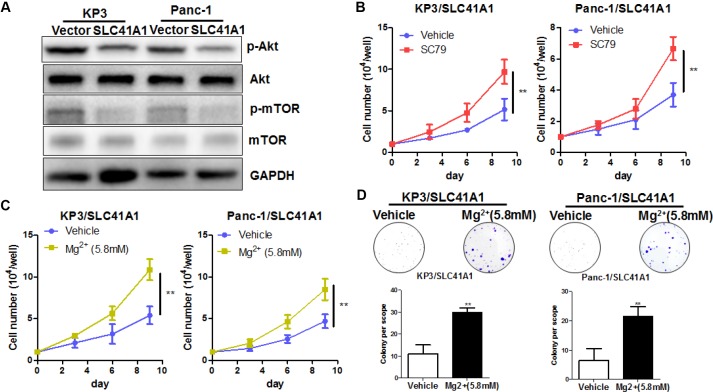
**Mg^2+^-dependent Akt/mTOR inhibition mediates the proliferation inhibitory effects of SLC41A1 in PDAC.** (**A**) Overexpression of SLC41A1 reduced the activation of Akt/mTOR signalling. (**B**) Treatment with the Akt activator SC79 partially recovered the cellular proliferation inhibited by SLC41A1. (**C**) Mg2+ supplementation abrogated the proliferation inhibition by SLC41A1. (**D**) Mg2+supplementation abrogated the inhibition of colony formation by SLC41A1.

**Figure 6:**
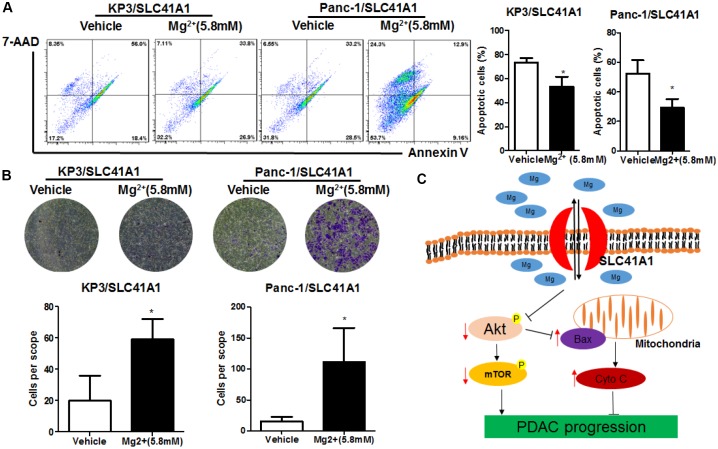
**Mg^2+^-dependent Akt/mTOR inhibition mediates the apoptosis induction and invasion suppression effects of SLC41A1 in PDAC.** (**A**) Mg^2+^ supplementation abrogated apoptosis induction by SLC41A1. (**B**) Mg^2+^supplementation abrogated the inhibition of invasion by SLC41A1. (**C**) Overall mechanisms underlying the anti-tumour effects of SLC41A1 in PDAC.

The presence of SLC41A1 across the cellular membrane mediates the efflux of intracellular Mg^2+^ [27]. Reduced cellular Mg^2+^ has been shown to inhibit intracellular signalling pathways including Akt/mTOR [28]. To determine whether the tumour-suppressive effects of SLC41A1 were associated with its function as an Mg^2+^ transporter, we evaluated the activity of Akt/mTOR in SLC41A1-overexpressing PDAC cells and found that the overexpression of SLC41A1 significantly inhibited Akt/mTOR activity (Figure 5A). This inactivation was correlated with the tumour-suppressive function of SLC41A1, as evidenced by the observation that recovery of Akt activity with SC79 (10 μg/mL) markedly abrogated the inhibition of PDAC cell proliferation by SLC41A1 overexpression (Figure 5B). Restoration of cell proliferation and colony formation was also observed when SLC41A1-overexpressing PDAC cells were supplemented with additional Mg^2+^ to compensate the ion efflux (Figure 5C, 5D). Mechanistically, this recovery may be associated with the reduced cell apoptosis of SLC41A1-overexpressing PDAC cells in the presence of additional Mg^2+^ (Figure 6A). Also, the invasiveness of SLC41A1-overexpressing PDAC cells was restored upon addition of Mg^2+^ (Figure 6B). These observations demonstrated that SLC41A1 may function as a tumour suppressor by regulating the activity of Mg^2+-^associated signalling pathways in PDAC cells.

## DISCUSSION

Although SLC proteins have been postulated to play an essential role in the progression and treatment of human cancers, their expression pattern and function in some cancers, including PDAC, have rarely been studied. In this study, we extracted and screened data from clinical samples to identify possible SLC proteins that are correlated with the clinical outcome of PDAC. Among the SLC proteins identified, SLC41A1 was found to be clinically relevant across different databases. SLC41A1, which was initially identified as the homology to the bacterial MgtE family of Mg^2+^ transporters [29], mediates Mg^2+^ efflux in eukaryotic cells [30]. It is ubiquitously expressed in several tissues and is upregulated in some tissues with Mg^2+^ deficiency [31]. Efflux of Mg^2+^ by SLC41A1 is strictly Na^+^-dependent [32]. SLC41A1 has multiple physiological functions. It was immediately and transiently downregulated by exercise, which was not reversed in the presence of Mg^2+^ [33]. SLC41A1 is overexpressed in the placenta of preeclamptic women and is responsible for changes in Mg^2+^ homeostasis in the development of preeclampsia [34]. Also, it has been proposed that SLC41A1 may be dysregulated under pathological conditions. For example, in patients with Parkinson’s disease from China, Taiwan, and Iran, the SLC41A1 gene was found to have different variants, which might be associated with its reduced expression and loss of function [20, 21, 23]. Also, the SLC41A1 variant might predict the risk of Parkinson’s disease in the Chinese population [22]. Furthermore, in damaged neurons, SLC41A1 was significantly reduced, which could be reversed by Mg^2+^, indicating its neuroprotection role [35]. Mutations in SLC41A1 were also observed in nephronophthisis, which resulted in the in-frame deletion of a transmembrane helix. Mutated SLC41A1 was not able to maintain renal Mg^2+^ homeostasis, resulting in tubular defects and nephronophthisis-like phenotypes [36]. The role of SLC41A1 in cancer has not yet been systematically evaluated. In this study, we found that SLC41A1 overexpression in PDAC cells inhibited the *in vitro* proliferation and *in vivo* tumour growth, as a result of the apoptotic-like phenotype of SLC41A1-overexpressing cells. This apoptotic phenotype could be attributed to disruption of Mg^2+^ homeostasis in PDAC cells via elimination of intracellular Mg^2+^. Interestingly, a recent analysis in patients with head and neck cancer undergoing cisplatin chemotherapy showed that SLC41A1 level was correlated with serum levels of Mg^2+^, suggesting that SLC41A1 might undergo Mg^2+^ extrusion from tumour tissues [37]. The findings from our study support the tumour-suppressive role of SLC41A1 in PDAC.

We observed that the tumour-suppressive activity of SLC41A1 appeared to be associated with its function as an Mg^2+^ transporter. A previous study showed that SLC41A1 mediated the regulation between Mg^2+^ concentration and mineralisation during the osteogenic differentiation of mesenchymal stromal cells [18], and reduced Mg^2+^ efflux was responsible for the prevention of cardiac fibrosis when SLC41A1 was knockdown [38]. We observed the partial recovery of the proliferation and invasion of PDAC cells after supplementation with a high concentration of Mg^2+^ to SLC41A1-overexpressing cells. Because the addition of Mg^2+^ into culture medium may saturate the Mg^2+^ concentration, Mg^2+^ efflux by SLC41A1 may be compromised, leading to maintenance of intracellular Mg^2+^ concentration. This led to partial abrogation of the anti-tumour effects of SLC41A1 overexpression in PDAC. Indeed, the role of Mg^2+^ in cell apoptosis has not been previously studied. High extracellular Mg^2+^ might inhibit apoptosis induced by endoplasmic reticulum stress inducers by increasing intracellular Mg^2+^ [39]. These observations were further supported by the fact that deletion of the Mg^2+^ influx transporter TRPM7 triggered apoptosis in bladder cancer [40]. Chronic dietary deficiency of Mg^2+^ might cause cardiac apoptosis in the rat heart [41]. Taken together, these studies indicate that maintaining intracellular Mg^2+^ homeostasis is crucial for the survival of cancer cells. The overexpression of SLC41A1 suppressed Akt/mTOR activity and induced Bax-associated MMP loss in PDAC cells. It has been previously shown that Mg^2+^ treatment could increase intracellular Mg^2+^ concentrations and activate Akt phosphorylation [42], whereas decrease of intracellular Mg^2+^ was observed during the mitochondrial apoptosis of colon cancer cells [43]. Mg^2+^ was a necessary factor for stabilisation of the mitochondrial membrane [44], possibly due to the maintenance of intracellular Mg^2+^/calcium (Ca^2+^) homeostasis [45]. Indeed, a previous study showed that Mg^2+^ could inhibit the uptake of Ca^2+^ by mitochondria, which was the initiating factor of MMP loss and Cytochrome C release [46]. The uptake of Ca^2+^ by mitochondria involved a contribution from Bax, which guided intracellular Ca^2+^ fluxes from the endoplasmic reticulum to mitochondria [47]. As Bax expression was suppressed by Akt/mTOR pathway [48], SLC41A1-suppressed Akt/mTOR signalling might activate Bax expression and subsequent cellular events. In such cases, the anti-tumour mechanism of SLC41A1 was proposed to be Mg^2+^-dependent, with Mg^2+^ depletion by SLC41A1 overexpression resulting in Akt/mTOR inhibition and subsequent induction of Bax expression, triggering loss of membrane integrity and release of Cytochrome C to activate caspase-dependent apoptosis in PDAC (Figure 6C).

## MATERIALS AND METHODS

### Chemicals, antibodies, and reagents

Crystal violet, the caspase inhibitor Z-VAD-FMK, and the Akt activator SC79 were purchased from Sigma-Aldrich (St. Louis, MO). JC-1 dye was purchased from Life Technologies (Carlsbad, CA). A non-viral plasmid expressing SLC41A1 open reading frame (ORF) was obtained from Origene Technologies (Rockville, MD). Scrambled negative control small interfering RNA (siRNA) and siRNA against Bcl-2-associated X protein (Bax) were purchased from Santa Cruz Biotechnology (Dallas, TX). Rabbit polyclonal antibody against SLC41A1 (#ab83701) was obtained from Abcam (Cambridge, UK). Rabbit monoclonal antibodies against Akt (#4685), phospho-Akt (p-Akt, Ser473, #4060), mechanistic target of rapamycin (mTOR, #2983), p-mTOR (Ser2448, #5536), Bax (#5023), cytochrome c (#4280), cleaved caspase-3 (#9664), and GAPDH (#5174) were purchased from Cell Signaling Technology (Danvers, MA).

### Cell lines and cell culture

The KP3 human pancreatic adenocarcinoma cell line expressing luciferase reporter gene was obtained from the Japanese Collection of Research Bioresources Cell Bank (Tokyo, Japan). Panc-1 and BxPC3 cell lines and the normal pancreatic ductal epithelial cell line hTERT-HPNE were obtained from the American Type Culture Collection (Manassas, VA). KP3, Panc-1, and BxPc3 cells were cultured in Dulbecco’s modified Eagle medium (DMEM, 4.5 g/L glucose; Thermo Fisher Scientific, Waltham, MA) supplemented with 10% fetal bovine serum (FBS) and 1% penicillin/streptomycin. hTERT-HPNE cells were cultured with a mixture of 75% DMEM (no glucose; Thermo Fisher Scientific), and 25% Medium M3 Base (InCell, Frisco, TX) supplemented with 5% FBS, 10 ng/mL human recombinant epidermal growth factor, 1g/L glucose, and 750 ng/mL puromycin. All cells were maintained under humidified conditions (5% CO_2_, 37°C).

### Cell proliferation and colony formation assays

Cell proliferation was measured by cell count. In brief, 1 × 10^4^ cells were seeded in a 6-well tissue culture plate. The whole cell population was collected after 3-, 6-, and 9-day incubations and cells were counted using a hemocytometer. For the colony formation assay, 1 × 10^4^ cells were seeded in a 6-well tissue culture plate and incubated for 12 days. Then, cells were fixed in 4% paraformaldehyde followed by staining with crystal violet. Images of the colonies were taken after washing and air drying. All experiments were conducted in triplicate.

### Matrigel invasion chamber assay

Matrigel matrix (BD Bioscience, Franklin Lakes, NJ) was diluted with cold phosphate-buffered saline (PBS, 1:4, v/v). Then, 100 μL diluted Matrigel was coated on the chamber insert (pore size: 8 μm; Corning, Corning, NY). A density of 1 × 10^5^ cells was seeded on top of the Matrigel matrix in serum-free DMEM, and the receiving chamber was filled with DMEM containing FBS. At 36 h post-seeding, the Matrigel matrix with non-invading cells was removed with cotton swabs. Cells at the basolateral membrane were fixed in 4% paraformaldehyde followed by staining with crystal violet. Images of invaded cells were taken after washing and air-drying. All experiments were conducted in triplicate.

### Flow cytometry

Detection of cell apoptosis was conducted with the Apoptosis Assay Kit I (BD Biosciences). In brief, cells were collected with trypsinisation followed by Annexin V-PE and 7-ADD staining in calcium-containing binding buffer for 10 min. Cells were then washed, re-suspended in PBS, and subjected to flow cytometry (BD FACSCanto II; BD Biosciences). Cells that were Annexin V-positive or Annexin V/7-AAD double-positive were considered apoptotic. For measurements of the mitochondrial membrane potential (MMP), cells in a 6-well tissue culture plate were stained with JC-I (10 μM) for 40 min in the dark. Then cells were trypsinised and washed with PBS before flow cytometry (BD FACSCanto II; BD Biosciences). Red fluorescence indicated an intact mitochondrial membrane, whereas an increase in green fluorescence suggested loss of the MMP.

### Quantitative real-time PCR

Total RNA was extracted with the RNeasy Kit (Qiagen, Hilden, Germany) and the first-strand cDNA was synthesised with a cDNA Synthesis Kit (Takara, Tokyo, Japan). Quantitative real-time PCR was performed with SYBR Green Master Mix (Takara) on the Lightcycler 480 platform (Roche, Indianapolis, IN). Primer sequences were as follows: forward: 5′-TGGAAGAAGATGGGCTGAG-3′ and reverse: 5′-GTGTCCCGAAGGAGGTTTA-3’ for human Bax; forward: 5′- AGAAGGATTCCTATGTGGGCG-3′ and reverse: 5′- CATGTCGTCCCAGTTGGTGAC-3′ for human β-actin. Bax expression was normalised by β-actin [13].

### Immunoblotting

Total protein was collected by extraction in RIPA buffer. The same amount of protein was separated by electrophoresis and then transferred to a polyvinylidene difluoride membrane. The membrane was blocked in 5% bovine serum albumin in Tris-buffered saline with Tween 20 buffer. Primary antibody incubation was conducted overnight at 4°C followed by incubation with the appropriate secondary antibody incubation at room temperature for 2 h. Chemiluminescent imaging was done with a gel imaging system (Bio-Rad Laboratories, Hercules, CA) using enhanced chemiluminescence as the substrate (GE Healthcare, Chicago, IL).

### Orthotopic pancreatic tumour model

Animal experiments protocols were reviewed and approved by the Ethics Committee of the Department of Laboratory Animal Science, Fudan University (No. LASFDI-20140238A091; Shanghai, China). Establishment of an orthotopic pancreatic tumour model was conducted according to previously published protocol with minor modifications [49]. In brief, 2 × 10^6^ KP3 cells expressing the luciferase reporter were gently mixed with Matrigel matrix (1:1, v/v). The entire pancreatic body and spleen were exposed, and 20 μL cell suspension was slowly injected into the pancreas head. Then, the pancreas and spleen were placed back in the mice, and the abdominal muscle and skin layers were closed. At 1 week post-implantation, mice were injected with 30 mg/kg D-luciferin under anaesthesia. Orthotopic tumour size was measured using an *in the vivo* imaging system (Xenogen Corp., Alameda, CA), and images were taken weekly for 4 weeks. At the end of the experiment, mice were sacrificed with pentobarbital overdose (200 mg/kg). Tumours were dissected from tumour-bearing mice.

### Human tissues

Six pairs of human PDAC samples with adjacent non-tumour tissues were collected from the tissue bank of the Department of Oncology, Shanghai Medical College, Fudan University. The protocol was approved by the ethics committee of the Department of Oncology, Fudan University (Ref. No. 050432-4-1212B).

### Statistical analysis

Experiments were conducted in triplicate. Data are presented as the mean ± standard deviation. The Student’s *t-*test was performed and p < 0.05 was considered statistically significant.

## CONCLUSIONS

We found that SLC41A1 has tumour-suppressive functions in PDAC. SLC41A1 was found to be downregulated in PDAC during disease progression and was positively correlated with the OS and disease-free survival of PDAC patients. Overexpression of SLC41A1 reduced tumour growth in an orthotopic mouse model of PDAC and decreased the *in vitro* proliferation of PDAC cells, which might be due to a reduction in the MMP, and subsequent release of Cytochrome C to trigger caspase-dependent apoptosis. The apoptotic-prone phenotype of SLC41A1-overexpressing PDAC cells might be dependent upon Mg^2+^ depletion-induced suppression of Akt/mTOR signalling and Bax expression. The results of this study suggest that SLC41A1 may be a novel therapeutic target for PDAC.

## Supplementary Material

Supplementary Figures
