# Supplementary material for: Magnesium transporter protein solute carrier family 41 member 1 suppresses human pancreatic ductal adenocarcinoma through magnesium-dependent Akt/mTOR inhibition and bax-associated mitochondrial apoptosis
Source: Aging (Albany NY). 2019 May 8;11(9):2681–98. doi: 10.18632/aging.101940 (PMC6535063; doi:10.18632/aging.101940)
Supplement: Supplementary Figures [file aging-11-101940-s001.pdf]

SUPPLEMENTARY FIGURES

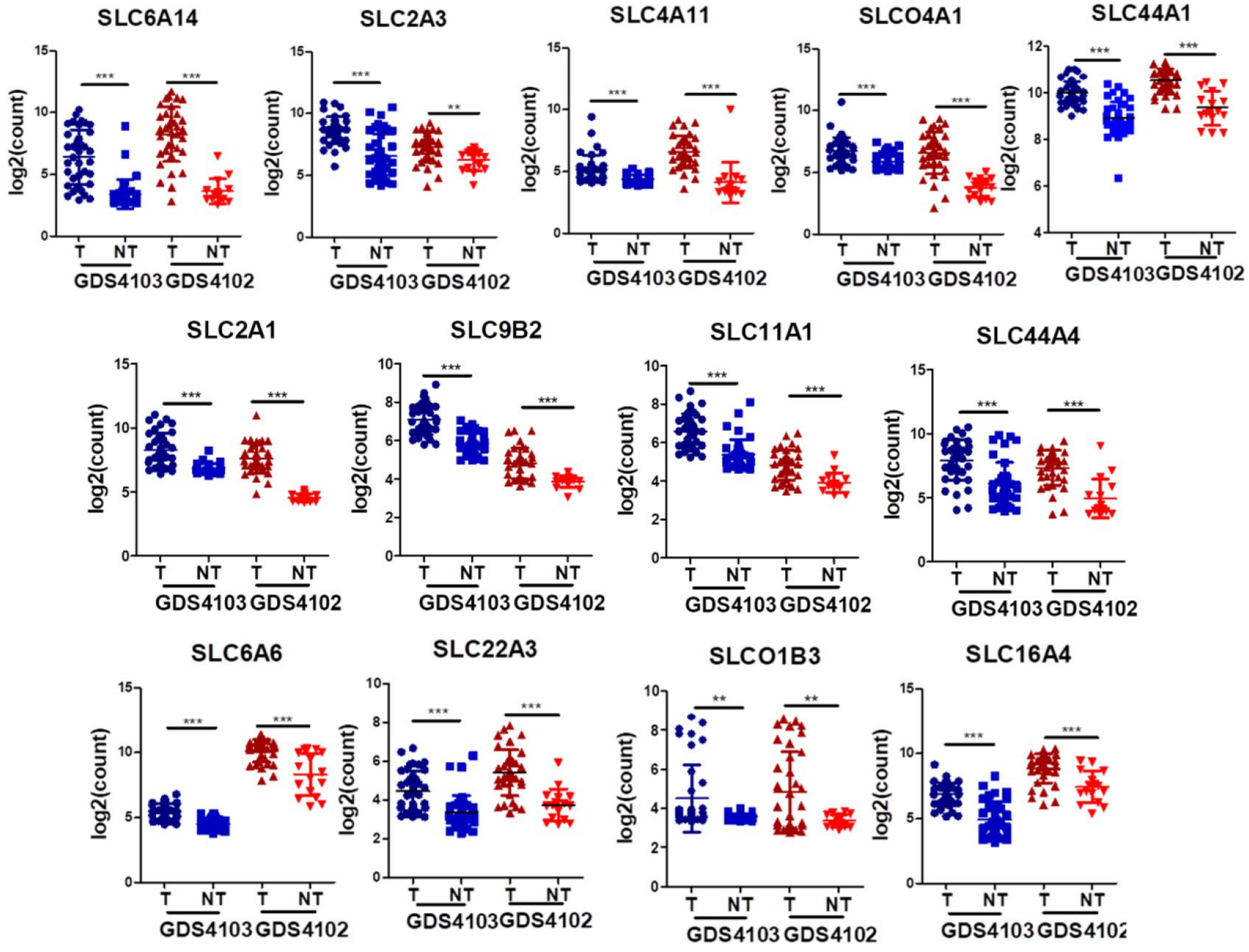

Supplementary Figure 1. SLC family of genes that were overexpressed in PDAC. *p < 0.05; **p < 0.01; ***p < 0.001.

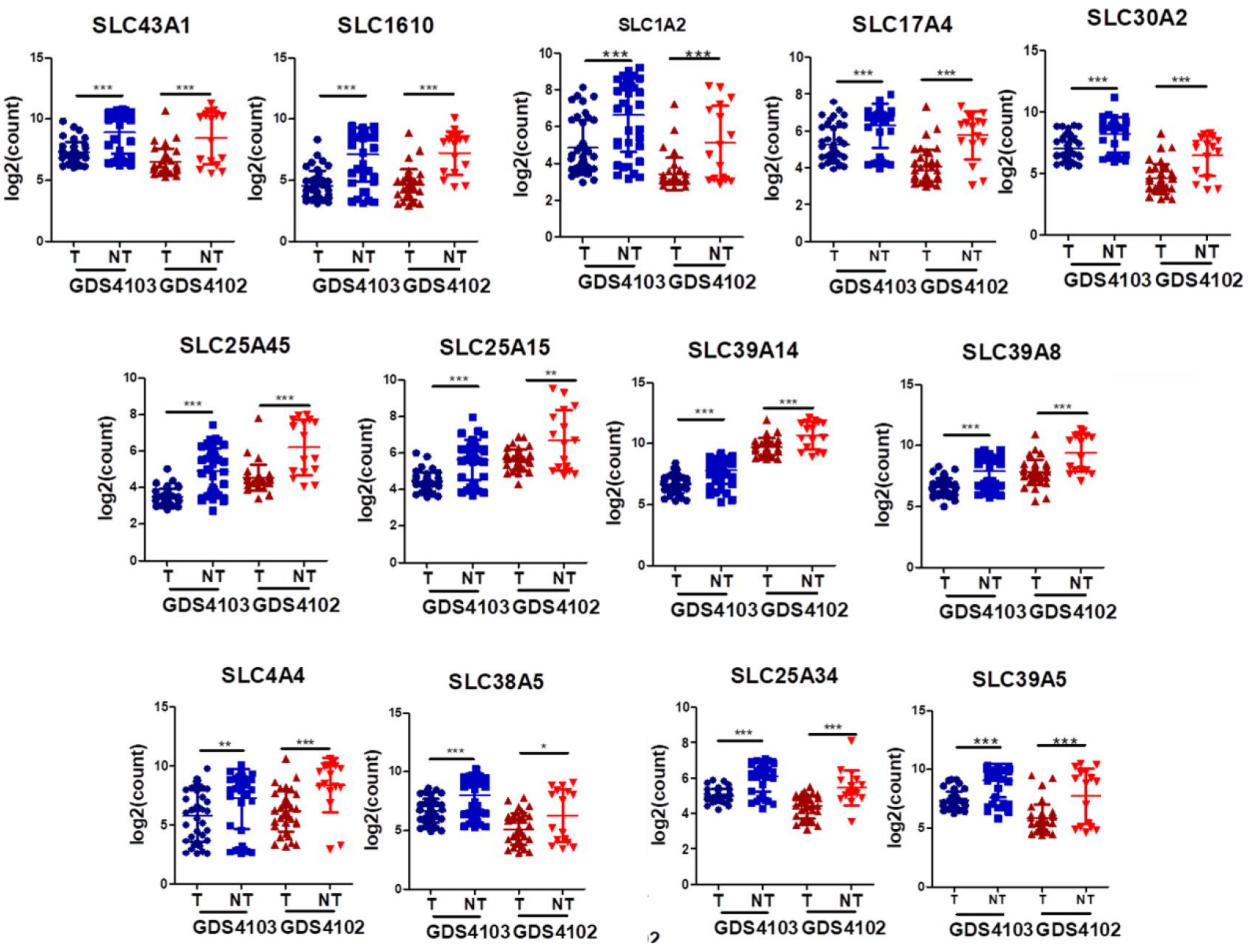

Supplementary Figure 2. SLC family of genes that were downregulated in PDAC. *p < 0.05; **p < 0.01; ***p < 0.001.

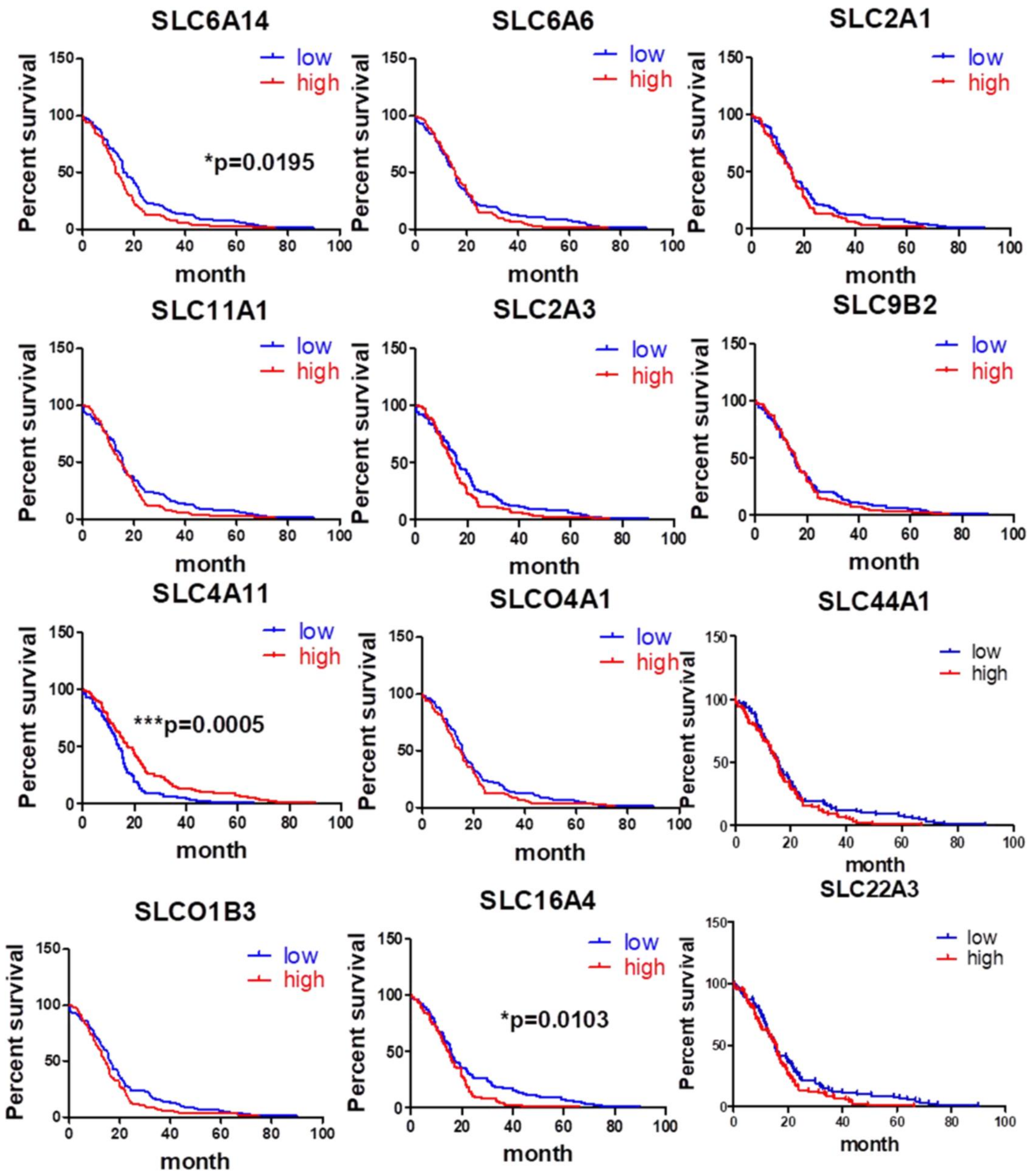

Supplementary Figure 3. Overall survival curve of SLC proteins overexpressing in PDAC. *p < 0.05; **p < 0.01.

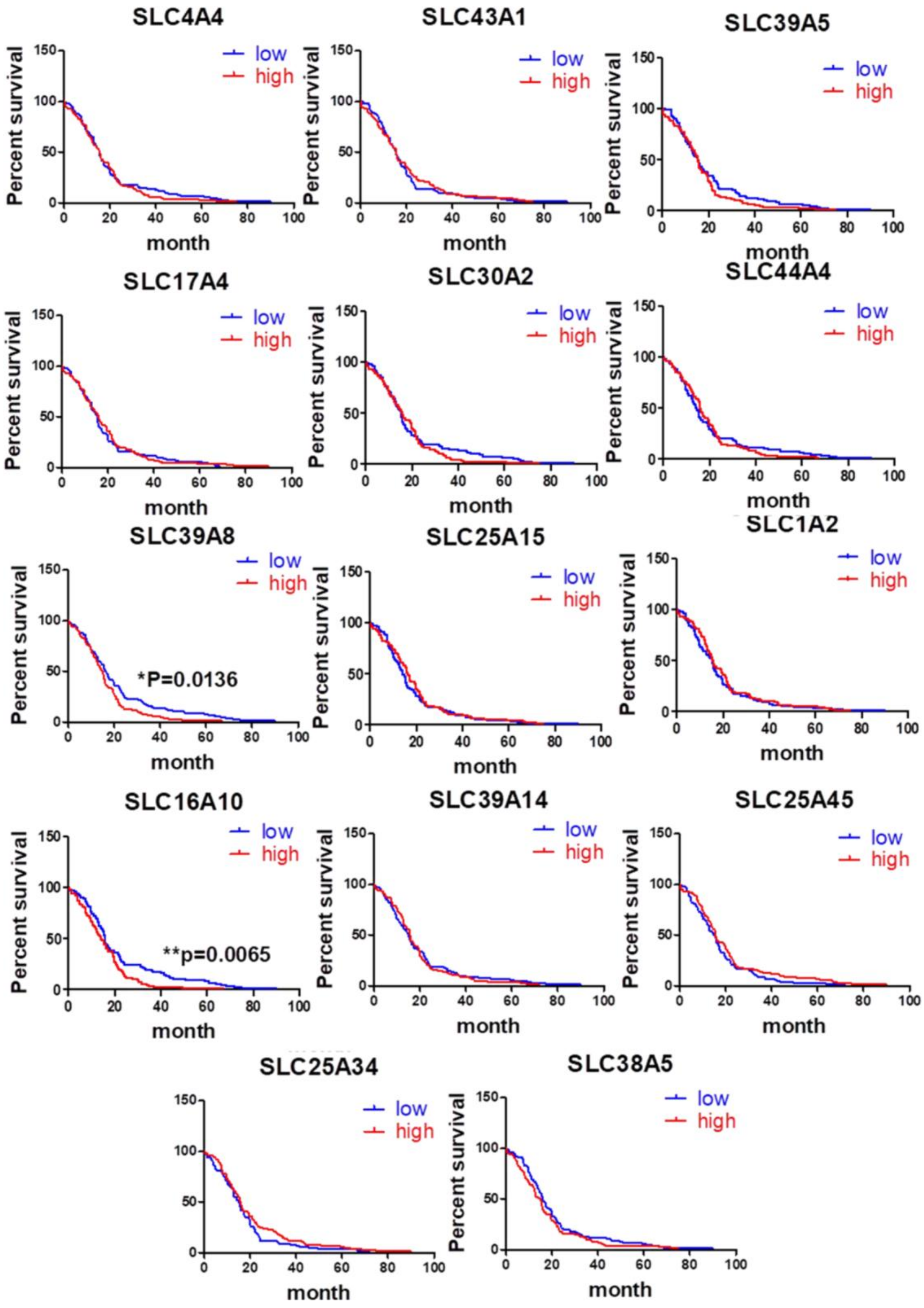

Supplementary Figure 4. Overall survival curve of SLC proteins down-regulated in PDAC. *p < 0.05; **p < 0.01.

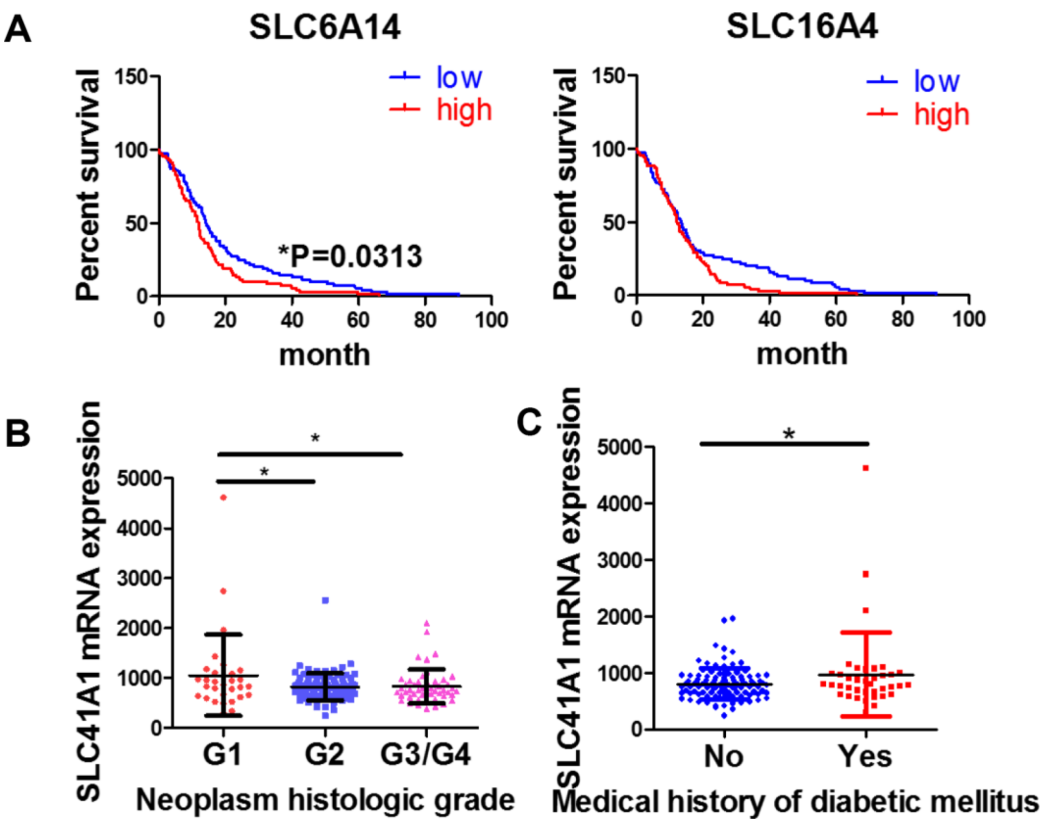

Supplementary Figure 5. Correlation of SLC protein expression with progression-free survival and disease characteristics of PDAC patients. (**A**) Disease-free survival curve of patients with differential SLC protein expression and corresponding progression-free survival. (**B**) Association of SLC41A1 expression with tumour stage progression. (**C**) Association of SLC41A1 expression with medical history of diabetes. *p < 0.05; **p < 0.01.
